# Mutational burden and potential oligogenic model of *TBX6*‐mediated genes in congenital scoliosis

**DOI:** 10.1002/mgg3.1453

**Published:** 2020-08-20

**Authors:** Yang Yang, Sen Zhao, Yuanqiang Zhang, Shengru Wang, Jiashen Shao, Bowen Liu, Yaqi Li, Zihui Yan, Yuchen Niu, Xiaoxin Li, Lianlei Wang, Yongyu Ye, Xisheng Weng, Zhihong Wu, Deciphering Disorders Involving Scoliosis and COmorbidities (DISCO) study, Jianguo Zhang, Nan Wu

**Affiliations:** ^1^ Department of Orthopedic Surgery Peking Union Medical College Hospital Peking Union Medical College and Chinese Academy of Medical Sciences Beijing China; ^2^ Beijing Key Laboratory for Genetic Research of Skeletal Deformity Beijing China; ^3^ Medical Research Center Peking Union Medical College Hospital Peking Union Medical College and Chinese Academy of Medical Sciences Beijing China; ^4^ Department of Orthopedic Surgery First Affiliated Hospital of Sun Yat‐sen University Sun Yat‐sen University Guangzhou China; ^5^ Key Laboratory of Big Data for Spinal Deformities Chinese Academy of Medical Sciences China

**Keywords:** congenital scoliosis, exome sequencing, mutational burden, *TBX6*‐mediated genes

## Abstract

**Background:**

Congenital scoliosis (CS) is a spinal deformity due to vertebral malformations. Although insufficiency of *TBX6* dosage contributes to a substantial proportion of CS, the molecular etiology for the majority of CS remains largely unknown. *TBX6*‐mediated genes involved in the process of somitogenesis represent promising candidates.

**Methods:**

Individuals affected with CS and without a positive genetic finding were referred to this study. Proband‐only exome sequencing (ES) were performed on the recruited individuals, followed by analysis of *TBX6*‐mediated candidate genes, namely *MEOX1*, *MEOX2*, *MESP2*, *MYOD1*, *MYF5*, *RIPPLY1*, and *RIPPLY2*.

**Results:**

A total of 584 patients with CS of unknown molecular etiology were recruited. After ES analysis, protein‐truncating variants in *RIPPLY1* and *MYF5* were identified from two individuals, respectively. In addition, we identified five deleterious missense variants (*MYOD1*, *n* = 4; *RIPPLY2*, *n* = 1) in *TBX6*‐mediated genes. We observed a significant mutational burden of *MYOD1* in CS (*p* = 0.032) compared with the in‐house controls (*n* = 1854). Moreover, a potential oligogenic disease‐causing mode was proposed based on the observed mutational co‐existence of *MYOD1*/*MEOX1* and *MYOD1*/*RIPPLY1*.

**Conclusion:**

Our study characterized the mutational spectrum of *TBX6*‐mediated genes, prioritized core candidate genes/variants, and provided insight into a potential oligogenic disease‐causing mode in CS.

## Graphical abstract

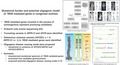

## 1 INTRODUCTION

Congenital scoliosis (CS) is a spinal deformity caused by malformations of vertebrae, which include the failure of formation (CS type I), failure of segmentation (CS type II), or a combination of the two (CS type III) (Hedequist & Emans, 2007). With an incidence of 0.5–1 per 1000 live births, CS is a major contributor to childhood and adolescent disability (Shen, Wang, Liu, Xue, & Qiu, 2013).

In our previous studies, we found that *TBX6* gene contributes to about 10% of CS patients with a compound inheritance disease‐causing mode, that is, a *TBX6* null mutation or 16p11.2 deletion in trans with a common T‐C‐A (rs2289292, rs3809624, and rs3809627) haplotype (Liu et al., 2019; Wu et al., 2015; Yang et al., 2019). We defined this entity of patients as *TBX6*‐associated congenital scoliosis (TACS) (Liu et al., 2019). In addition to null mutations and copy number deletions, missense variants in *TBX6* which lead to a functional null effect also lead to TACS in combination with the risk T‐C‐A allele (Chen et al., 2020). In contrast to this mutation + polymorphism combination, biallelic loss‐of‐function or dominant‐negative mutations in *TBX6* cause Spondylocostal Dysostosis (MIM#122600), a severe skeletal dysplasia syndrome characterized by multiple segmentation defects of the spine and costal dysplasia(Sparrow et al., 2013). Although *TBX6* mutations contribute to a substantial proportion of CS, the molecular etiology for the majority of CS remains largely unknown, and more candidate genes/loci warrant investigation.

During the process of somite segment determination, *TBX6* activates mesoderm posterior 2 (MESP2) (Morimoto, Takahashi, Endo, & Saga, 2005; Oginuma, Niwa, Chapman, & Saga, 2008; Takahashi et al., 2000; Yasuhiko et al., 2006). *MESP2* then promotes the formation of somite boundary via activation of *RIPPLY1*/*RIPPLY2*, which negatively regulates *TBX6* expression (Nakajima, Morimoto, Takahashi, Koseki, & Saga, 2006; Takahashi et al., 2010). *MESP2* also promotes the expression of *MYOD1* (Bondue et al., 2008; Stamataki, Kastrinaki, Mankoo, Pachnis, & Karagogeos, 2001; Windner et al., 2015), which then activates several pre‐myogenic mesoderm factors essential for myogenesis and somitogenesis including *MYF5*, *MEOX1*, and *MEOX2* (Gianakopoulos et al., 2011; Stamataki et al., 2001).

Of these *TBX6*‐mediated genes, *RIPPLY2*, *MESP2*, and *MYF5* are associated with known Mendelian syndromes involving skeletal abnormalities. Similar with *TBX6*, biallelic loss‐of‐function variants in *RIPPLY2* or *MESP2* cause autosomal recessive Spondylocostal Dysostosis (MIM#616566 for *RIPPLY2* and MIM#608681 for *MESP2*). Recessive *MYF5* variants are recently reported to be associated with External Ophthalmoplegia, Rib, and Vertebral Anomalies (MIM#618155), whose spine phenotype highly resembles that of Spondylocostal Dysostosis (Di Gioia et al., 2018). Therefore, these *TBX6*‐mediated genes together represent a promising candidate gene set for CS. However, due to the milder symptom of CS compared with the above‐mentioned recessive Mendelian syndromes, the effect of the candidate variants in CS probably vary and their disease‐causing modes are presumably complicated.

To explore the mutational landscape of *TBX6*‐mediated genes in CS, and to give insight into the potential multi‐factorial disease‐causing mode, we performed exome sequencing (ES) on 584 individuals with congenital scoliosis without a prior molecular diagnosis, and then, studied both heterozygous variants and combinations of variant alleles.

## 2 MATERIALS AND METHODS

### 2.1 Ethical compliance

Approval for the study was obtained from the ethics committee at Peking Union Medical College Hospital (JS‐098). Written informed consent was provided by each participant.

### 2.2 Patient recruitment

We consecutively recruited individuals who are affected with CS in Peking Union Medical College Hospital from 2009 to 2016, as part of the Deciphering Disorders Involving Scoliosis and COmorbidities (DISCO) study (http://www.discostudy.org/).

### 2.3 Exome sequencing

Proband‐only exome sequencing was performed on patients (1) without a molecular diagnosis of TACS, that is, not caused by defects of *TBX6* gene; (2) without a molecular diagnosis by any other known CS‐causing gene. In brief, genomic DNA was extracted from the peripheral blood. Illumina paired‐end libraries were prepared from DNA samples and subjected to exome capture using one of four capture kits (xGEN targeted capture kit [IDT], seqcap pure capture kit [Nimblegen], VCRome SeqCap EZ Choice HGSC 96 Reactions [Roche], and All Exon V6+UTR r2 core design [91 Mb, Agilent]), depending on the time of enrollment in our cohort, followed by sequencing on an Illumina HiSeq 2000/4000 platform.

### 2.4 Variant calling and annotation

Raw data from exome sequencing were processed using PUMCH Pipeline (PUMP) (Wang et al., 2018; Zhao et al., 2020). GRCh37 or hg19 (GenBank accession number: GCA_000001405.1) was used as the reference sequence. HaplotypeCaller function of Genome Analysis Toolkit (GATK), version 3.4.0. was used for calling of single‐nucleotide variants and small insertion/deletions (indels). Combined Annotation Dependent Depletion (CADD) (Kircher et al., 2014) were used to predict the conservation and pathogenicity of the variants. Minor allele frequency (MAF) of each variant was obtained from public databases including the 1000 Genomes Project (http://www.internationalgenome.org/), the Exome variant server, NHLBI GO Exome Sequencing Project (ESP) (http://evs.gs.washington.edu/EVS/), the Exome Aggregation Consortium (ExAC) (http://exac.broadinstitute.org/), and the Genome Aggregation Database (gnomAD) (https://gnomad.broadinstitute.org/).

### 2.5 Analytic strategy

*TBX6*‐mediated genes were curated through literature review. Only genes regulated by *TBX6* during somitogenesis were selected, including *MEOX1*, *MEOX2*, *MESP2*, *MYOD1*, *MYF5*, *RIPPLY1*, and *RIPPLY2*. Variants in candidate genes were filtered against population databases. Because a MAF of 0.01 is generally used to distinguish a variant from a polymorphism, we define a rare variant as having a MAF lower than 0.01. Taken into consideration the disease incidence of congenital scoliosis (0.0005–0.001) (Shen et al., 2013), we suggest that a variant with MAF ≥0.001 should not be pathogenic in a dominant disease trait. Therefore, a MAF cutoff of 0.001 was used to filter for ultrarare variants. In our analysis, ultrarare variants (MAF ≤0.001) were selected for monogenic analysis and rare variants (MAF ≤0.01) were selected for potential oligogenic analysis. Protein‐truncating variants (including stop‐gain, frameshift, splice donor, splice acceptor, and start‐loss variant), predicted deleterious missense variants (CADD score ≥20) and in‐frame indels were prioritized. Sanger sequencing was performed on protein‐truncating variants, deleterious missense variants, and variants presenting an oligogenic disease‐causing mode.

### 2.6 In‐house controls

In‐house controls are consisted of 1854 unrelated individuals without apparent skeletal deformities. ES were performed on these individuals using the same protocol as employed in CS cases.

### 2.7 Statistics

R (version 3.6.1) was used for the statistical analysis. Fisher's Exact Test was used to compare the burden of deleterious missense variants with MAF ≤0.001 between cases and controls.

## 3 RESULTS

### 3.1 Mutational spectrum of ultrarare variants in *TBX6*‐mediated genes

**Figure 1:**
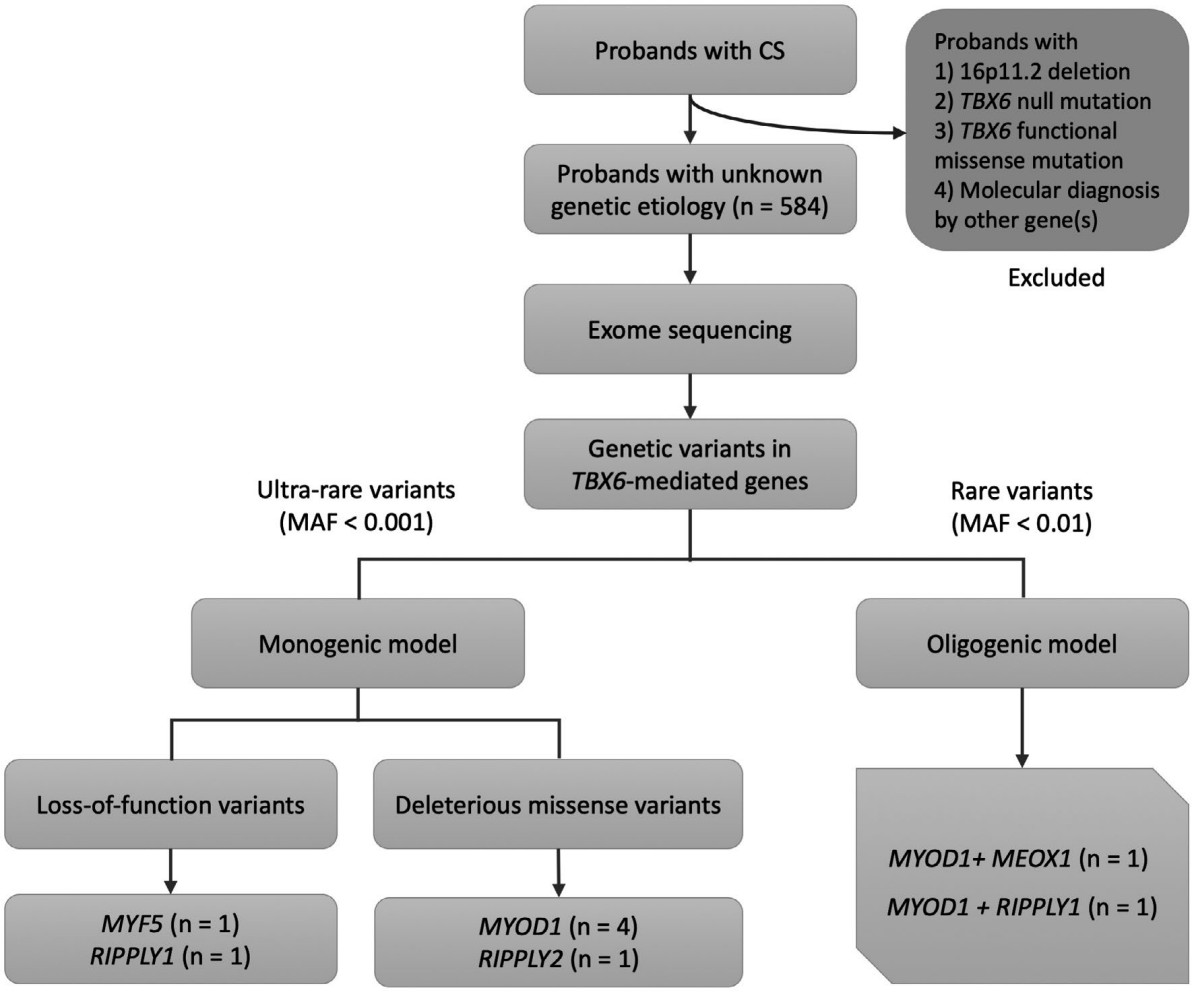
Workflow and main findings of the study

**Figure 2:**
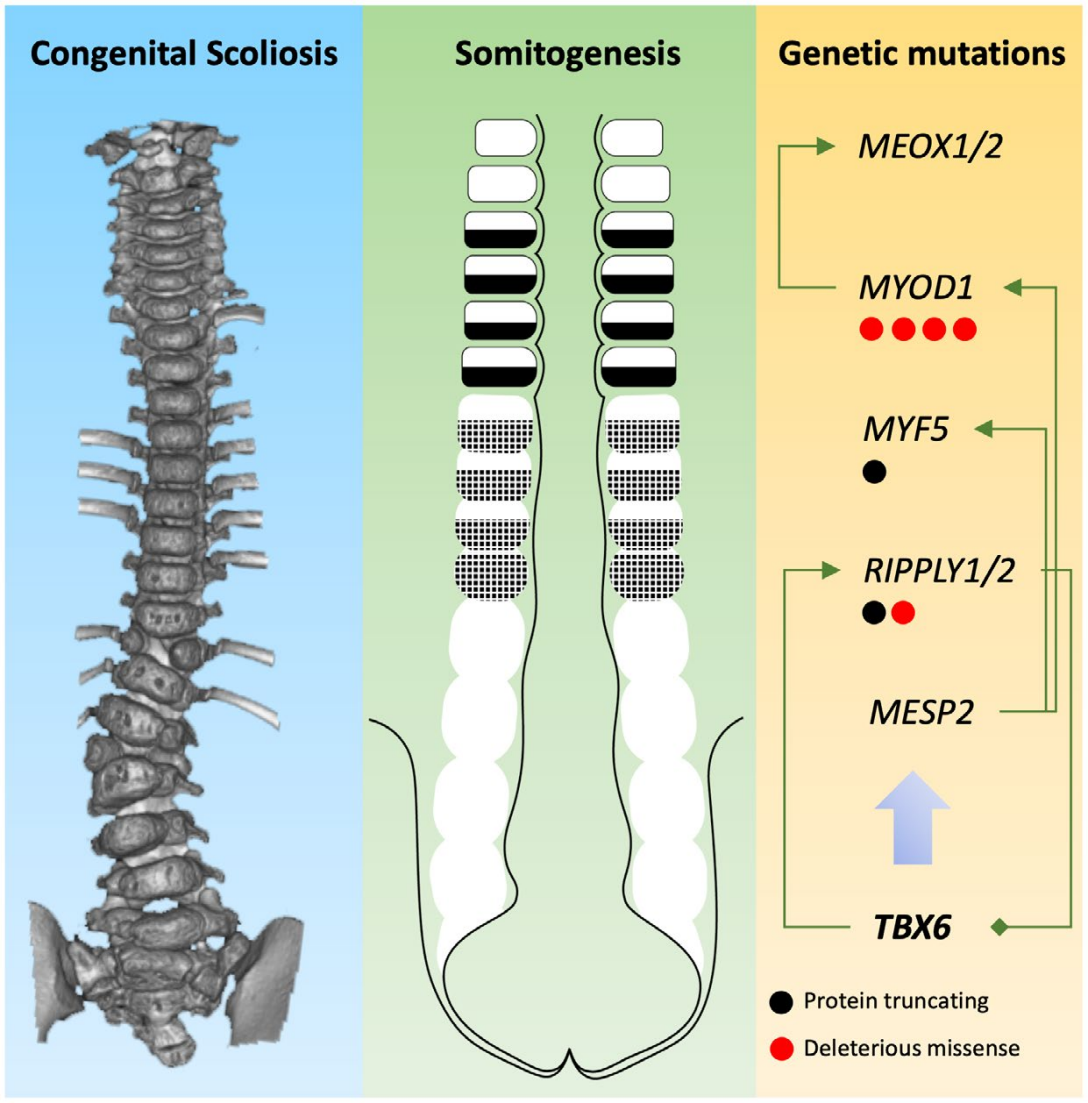
Developmental and molecular etiology of congenital scoliosis. The illustration demonstrates how genetic mutations identified in *TBX6*‐mediated genes may alter the process of somitogenesis and whereby cause congenital scoliosis. Arrows between gene symbols indicate activation. The diamond between *RIPPLY1*/*2* and *TBX6* indicates a negative feedback between the molecules

We recruited 584 individuals affected with CS that could not be explained by *TBX6* or other CS‐causing genes (Figure 1). After ES data processing and variant filtering, we identified a total of 28 ultrarare (MAF ≤0.001) variants in *TBX6*‐mediated genes (Figures 2 and 4). Of them, seven variants are protein‐truncating or predicted to be deleterious (CADD >15), presenting a tendency toward a significant mutational burden as compared with the in‐house controls (nine ultrarare deleterious variants in 1854 control samples, *p* = 0.08, Fisher's Exact Test).

### 3.2 Identification of protein‐truncating variants in *RIPPLY1* and *MYF5*

We first analyzed variants predicted to cause complete loss‐of‐function of the proteins. As a result, two protein‐truncating variants in *TBX6*‐mediated genes were identified from two patients (Table 1).

**Table 1:** Protein‐truncating and deleterious missense variants

Case ID	Zygosity	Chr	Pos	VAF	Mutation type	Gene	Variant	gnomAD‐MAF	gnomAD‐EAS‐MAF	ExAC‐MAF	ExAC‐EAS‐MAF	Gerp++	CADD	pLI
CSS161458	Hem	X	106145446	100	Splice acceptor	*RIPPLY1*	c.156‐1G>C	0	0	0	0	4.33	—	0.01
CSS160633	Het	12	81110981	48.39	Nonsense	*MYF5*	c.139C>T(p.Gln47Ter)	0	0	0	0	6.17	36	0
CSS161458	Het	11	17741793	50	Missense	*MYOD1*	c.464G>T(p.Arg155Leu)	0	0	0.000026	0.00036	4.74	35	0.15
CSS161565	Het	11	17741787	52	Missense	*MYOD1*	c.458C>G(p.Ala153Gly)	0	0	0.000026	0.00036	4.88	34	0.15
CSS161580	Het	11	17741915	52.63	Missense	*MYOD1*	c.586G>T(p.Asp196Tyr)	0	0	0	0	4.74	23	0.15
CSS170323	Het	11	17741959	53.49	Missense	*MYOD1*	c.630G>C(p.Met210Ile)	3.23E‐05	0.0006	0.000015	0.00017	4.55	22.2	0.15
CSS170010	Het	6	84567046	44.44	Missense	*RIPPLY2*	c.325A>T(p.Ile109Phe)	0	0	0	0	5.64	21.5	0.01

Note:

GRCh37 or hg19 (GenBank accession number: GCA_000001405.1) was used as the reference sequence.

Abbreviations: CADD, Combined Annotation Dependent Depletion; Chr, chromosome; EAS, East Asian population; MAF, minor allele frequency; pLI, probability of loss‐of‐function intolerance; Pos, position; VAF, variant allele frequency.

**Figure 3:**
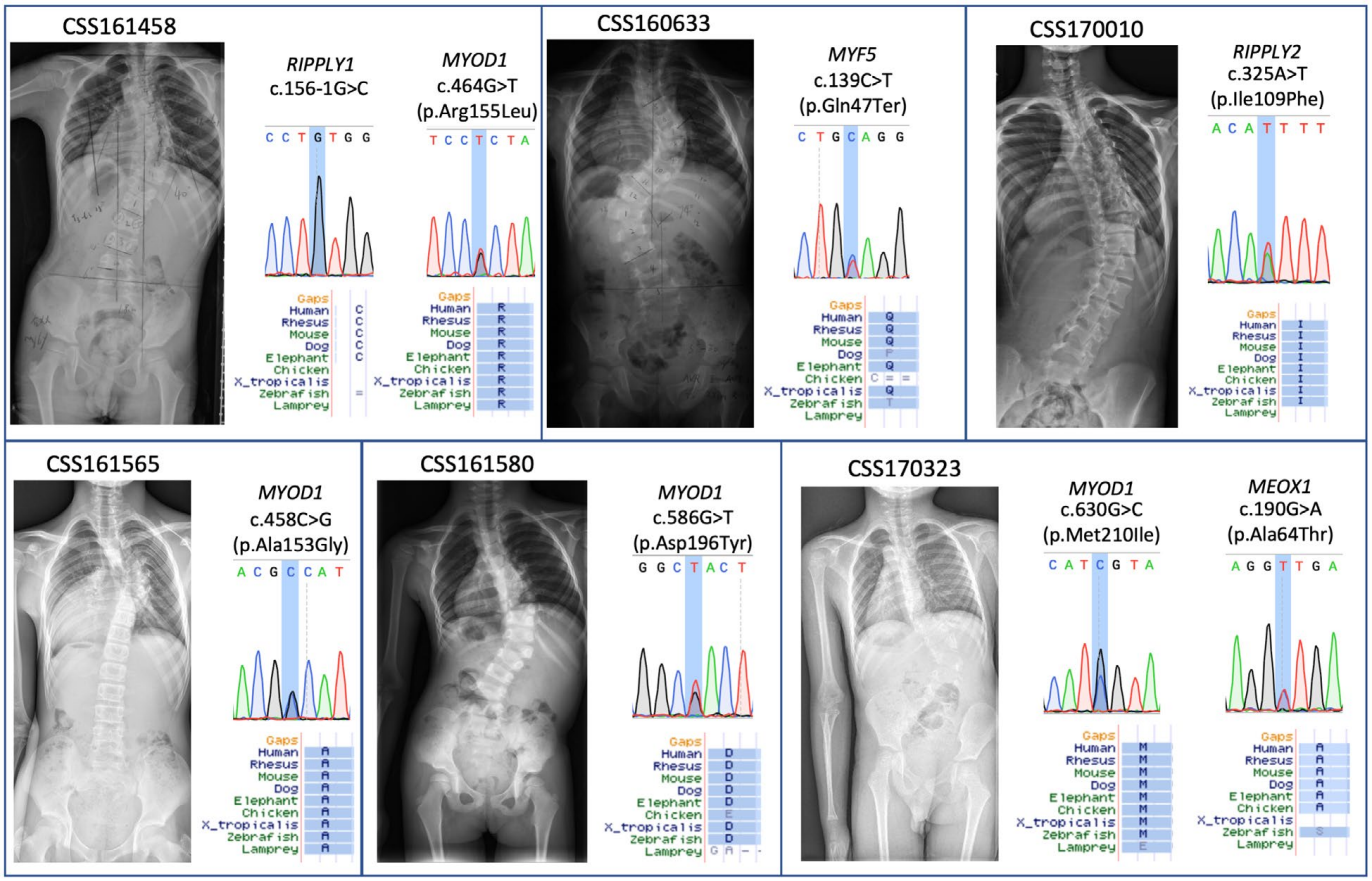
Images and mutational information of patients with protein‐truncating or deleterious missense variants. Spine X‐rays of six patients with truncating variants or deleterious missense variants are presented. Sanger sequencing results and residue conservation of these variants are also shown

A hemizygous splice‐donor variant c.156‐1G>C in *RIPPLY1* was identified in CSS161458, a 6‐year‐old boy affected with CS type II. CSS161458 had a right thoracic curve (magnitude of 40°, apex located at T9) with multiple segmentation failures from T8 to L1 (Figure 3). Although *RIPPLY1 *has not been associated with a human developmental disease, its paralog, *RIPPLY2*, causes Spondylocostal Dysostosis (MIM#616566) in an autosomal recessive mode. In addition, knock‐out of *Ripply1*/*Ripply2* in mouse results in disrupted somitogenesis and causes gross spinal deformity (J. Takahashi et al., 2010), suggesting the important role of *RIPPLY1* in the normal development of the spine. Our data suggest that truncating variants in *RIPPLY1*, in a hemizygous state, could be associated with vertebrae malformations in human.

A heterozygous stop‐gain variant in *MYF5* was identified in CSS160633, a 3‐year‐old boy affected with type I CS. He had two curves located at thoracic spine and lumbar spine, with wedge vertebrae located at apex region separately (Figure 3). Recently, biallelic frameshift and deleterious missense variants in *MYF5 *has been associated with External Ophthalmoplegia, Rib, and Vertebral Anomalies (MIM#618155). The spinal phenotypes of this syndrome include cervical and thoracic scoliosis, cervical fusions, clivus malformations, basilar invagination, and narrow disc spaces, which represents a more complex condition than that in our patient. Therefore, we propose that heterozygous loss‐of‐function of *MYF5* might be associated with a non‐syndromic form of CS.

### 3.3 Deleterious missense variants

In addition to protein‐truncating variants, we identified five deleterious missense variants (*n* = 4 for *MYOD1*, *n* = 1 for *RIPPLY2*) in *TBX6*‐mediated genes (Table 1).

**Figure 4:**
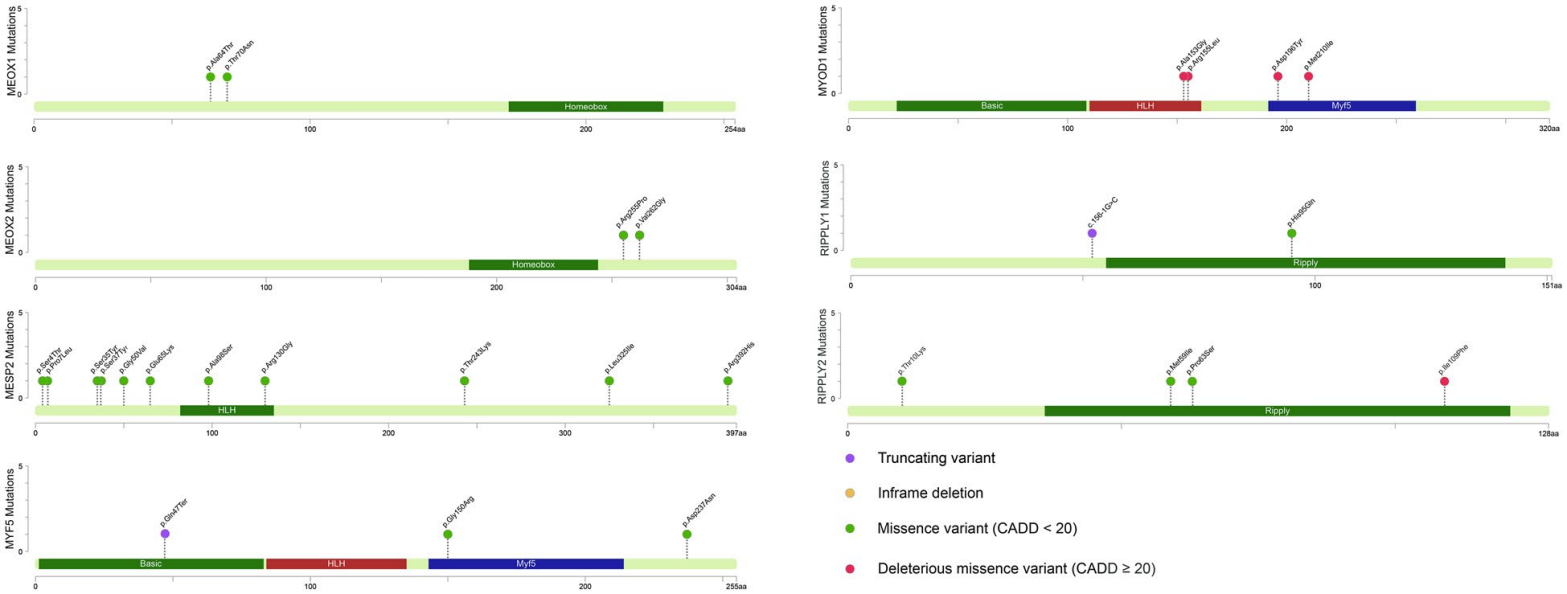
Mutational spectrum of the candidate genes in CS with unknown molecular etiology. Mapping of genetic variants in candidate genes to protein sequences annotated with functional domains

Notably, we observed an excess of ultrarare deleterious missense variants in *MYOD1* as compared with the in‐house control (4/584 in CS cases vs. 2/1854 in controls, *p* = 0.032, Fisher's Exact Test). These findings suggest the possible involvement of *MYOD1* in the pathogenesis of CS. *MYOD1* encodes an early transcriptional factor during somitogenesis, and is required for *MYF5* expression in the early mesoderm (Maguire, Isaacs, & Pownall, 2012). Intriguingly, two out of four deleterious missense variants in *MYF5* were adjacently located in the HLH domain (Figure 4), which is essential for the transcriptional activity of *MYF5* (Hamamori, Wu, Sartorelli, & Kedes, 1997). Therefore, these two variants c.464G>T(p.Arg155Leu) and c.458C>G(p.Ala153Gly) represent promising candidates in our analysis, and suggest the perturbation of the HLH domain as a potential etiology of CS.

### 3.4 Potential oligogenic disease‐causing mode

To give further insight into the complex molecular mechanism of CS, we analyzed variant combinations of *TBX6*‐mediated genes. As a result, we found two patients with a potential oligogenic inheritance (Table 2).

**Table 2:** Potential oligogenic variants identified in two probands

Case ID	Zygosity	Chr	Pos	VAF	Mutation type	Gene	Variant	gnomAD‐AF	gnomAD‐EAS‐AF	ExAC‐AF	ExAC‐EAS‐AF	Gerp++	CADD	pLI
CSS170323	Het	11	17741959	53.49	Missense	*MYOD1*	c.630G>C(p.Met210Ile)	3.23E‐05	0.0006	0.000015	0.00017	4.55	22.2	0.15
CSS170323	Het	17	41738713	50.43	Missense	*MEOX1*	c.190G>A(p.Ala64Thr)	3.24E‐05	0.0006	0.000075	0.001	2.64	7.499	0
CSS161458	Het	11	17741793	50	Missense	*MYOD1*	c.464G>T(p.Arg155Leu)	0	0	0.000026	0.00036	4.74	35	0.15
CSS161458	Hom	X	106145446	100	Splice acceptor	*RIPPLY1*	c.156‐1G>C	0	0	0	0	4.33	10.73	0.01

Note:

GRCh37 or hg19 (GenBank accession number: GCA_000001405.1) was used as the reference sequence.

Abbreviations: CADD, Combined Annotation Dependent Depletion; Chr, chromosome; EAS, East Asian population; MAF, minor allele frequency; pLI, probability of loss‐of‐function intolerance;Pos, position; VAF, variant allele frequency.

CSS170323 carries a heterozygous missense variant c.630G>C(p.Met210Ile) in *MYOD1* and a heterozygous missense variant c.190G>A(p.Ala64Thr) in *MEOX1* (Table 2). CSS170323 presented with L2 hemivertebra and fused ribs (the right 11th rib and 12th rib). During mesoderm development, the expression of *MEOX1* is increased by *MYOD1* (Gianakopoulos et al., 2011), suggesting that these two variant potentially result in the cumulative perturbation of *TBX6*‐mediated pathway.

CSS161458 had a heterozygous splicing variant c.156‐1G>C in *RIPPLY1*, as described above, and a heterozygous missense variant c.464G>T(p.Arg155Leu) in *MYOD1* was also identified. Although no direct interaction between *RIPPLY1* and *MYOD1 *has been reported, they may together dysregulate the *TBX6* pathway given the deleterious nature of both variants (Table 2).

## 4 DISCUSSION

In this study, we performed exome sequencing on 584 patients with CS and without a molecular diagnosis. Variants in seven *TBX6*‐mediated genes involved in somitogenesis were selected for analysis. Protein‐truncating variants, in‐frame indels and deleterious missense variants were prioritized. Potential oligenic disease‐causing modes were also identified.

The candidate gene strategy has been widely used in parallel sequencing studies on presumptive genetic disorders (Lin et al., 2020; Wu et al., 2019). The biological relevance and the rationality of the candidate gene set are critical for the success of this strategy. For example, focusing on genes encoding ion channels could promote the identification of novel genes in epilepsy (Epi & Epilepsy Phenome/Genome, 2017); the use of a systemic multiple candidate gene approach also explains a substantial proportion of the heritability in complex traits such as hypertension (Ji et al., 2017).

In our disease context of CS, the candidate gene set was selected based on a developmental biologic aspect: the skeletal system of a vertebrate embryo originates from the pre‐somitic mesoderm, which segments into somite during early embryo development. During this process, *TBX6* is expressed in the entire pre‐somitic mesoderm, and regulates a series of genes to enable the normal somitogenesis and subsequent development of the skeletal system (Oginuma et al., 2008). As *TBX6 *has been revealed as a core disease gene of CS (Chen et al., 2020; Liu et al., 2019; Wu et al., 2015), we conducted this study based on a hypothesis that *TBX6*‐mediated genes may as well contribute to CS. The rationality of this hypothesis is supported by disrupted spinal development in animal models depleted of the candidate genes (Takahashi et al., 2010; Windner et al., 2015), and by several autosomal recessive syndromes resulting in spinal malformation cause by these genes (Di Gioia et al., 2018; Karaca et al., 2015).

Due to the fact that CS represents a relatively milder phenotype spectrum than the autosomal recessive syndromes caused by biallelic loss‐of‐function of the candidate genes, the variant spectrum and disease‐causing mode of these genes in CS are assumed to be more complicated. More recently, there has been a surge of interest in digenic/oligogenic inheritance mode in complex disease (Schaffer, 2013). For example, mutational combination of *PCDH15* and *USH1G* have been identified in non‐syndromic hearing loss; oligogenic inheritance of three cardiomyopathy‐associated genes has been reported in a family with cardiac anomaly and supported by experimental evidence (Gifford et al., 2019). In our study, potential oligogenic inheritance mode were identified in two cases. Mutation combinations between *MYOD1*/*MEOX1* and *MYOD1*/*RIPPLY1* were observed. Given the relatively low probability of loss‐of‐function intolerance (pLI) of the candidate genes in the gnomAD database, a deleterious variant alone in any of these genes might not be disease causing. However, the combined effect of deleterious variants in multiple genes might synergistically lead to the disease. Although experimental validation of the oligogenic model still needs to be conducted, our data give insight into the complex disease‐causing mode of CS and suggest the combined effect of mutations in *TBX6*‐mediated genes as an important mechanism in the pathogenesis of CS.

In conclusion, our study characterized the mutational spectrum of *TBX6*‐mediated genes in CS, prioritized core candidate genes/variants, and provided insight into a potential oligogenic disease‐causing mode.

## CONFLICT OF INTEREST

The authors declare that they have no conflicts of interest.

## AUTHOR CONTRIBUTIONS

Jianguo Zhang, Nan Wu, Zhihong Wu, and Xisheng Weng conceived of the project and designed the study, Yang Yang, Sen Zhao, Yuanqiang Zhang, Shengru Wang, Jiashen Shao, Bowen Liu, Yaqi Li, and Zihui Yan collected and interpreted the data, Lianlei Wang, Yongyu Ye, Jiachen L, Hengqiang Z, Zihui Y, Zefu C, and Gang L conducted the statistical analysis. Yuchen Niu and Ziaoxin Li conducted the bioinformatic analyses. Yangyang, Yuanqiang Zhang, and Lianlei Wang recruited patients. Sen Zhao, Yangyang, and Nan Wu wrote the first draft of the manuscript, Jianguo Zhang, Zhihong Wu, and Xisheng Weng critically revised the work for important intellectual content.

## References

[mgg31453-bib-0001] Bondue, A. , Lapouge, G. , Paulissen, C. , Semeraro, C. , Iacovino, M. , Kyba, M. , & Blanpain, C. (2008). Mesp1 acts as a master regulator of multipotent cardiovascular progenitor specification. Cell Stem Cell, 3(1), 69–84. 10.1016/j.stem.2008.06.009 18593560

[mgg31453-bib-0002] Chen, W. , Lin, J. , Wang, L. , Li, X. , Zhao, S. , Liu, J. , … Wu, N. (2020). TBX6 missense variants expand the mutational spectrum in a non‐Mendelian inheritance disease. Human Mutation, 41(1), 182–195. 10.1002/humu.23907 31471994 PMC7061259

[mgg31453-bib-0003] Di Gioia, S. A. , Shaaban, S. , Tüysüz, B. , Elcioglu, N. H. , Chan, W.‐M. , Robson, C. D. , … Engle, E. C. (2018). Recessive MYF5 mutations cause external ophthalmoplegia, rib, and vertebral anomalies. American Journal of Human Genetics, 103(1), 115–124. 10.1016/j.ajhg.2018.05.003 29887215 PMC6035164

[mgg31453-bib-0004] Epi, K. c., & Epilepsy Phenome/Genome, P. (2017). Ultra‐rare genetic variation in common epilepsies: a case‐control sequencing study. The Lancet Neurology, 16(2), 135–143. 10.1016/S1474-4422(16)30359-3 28102150

[mgg31453-bib-0005] Gianakopoulos, P. J. , Mehta, V. , Voronova, A. , Cao, Y. I. , Yao, Z. , Coutu, J. , … Skerjanc, I. S. (2011). MyoD directly up‐regulates premyogenic mesoderm factors during induction of skeletal myogenesis in stem cells. Journal of Biological Chemistry, 286(4), 2517–2525. 10.1074/jbc.M110.163709 21078671 PMC3024746

[mgg31453-bib-0006] Gifford, C. A. , Ranade, S. S. , Samarakoon, R. , Salunga, H. T. , de Soysa, T. Y. , Huang, Y. U. , … Srivastava, D. (2019). Oligogenic inheritance of a human heart disease involving a genetic modifier. Science, 364(6443), 865–870. 10.1126/science.aat5056 31147515 PMC6557373

[mgg31453-bib-0007] Hamamori, Y. , Wu, H. Y. , Sartorelli, V. , & Kedes, L. (1997). The basic domain of myogenic basic helix‐loop‐helix (bHLH) proteins is the novel target for direct inhibition by another bHLH protein. Twist. Mol Cell Biol, 17(11), 6563–6573. 10.1128/mcb.17.11.6563 9343420 PMC232510

[mgg31453-bib-0008] Hedequist, D. , & Emans, J. (2007). Congenital scoliosis: A review and update. Journal of Pediatric Orthopedics, 27(1), 106–116. 10.1097/BPO.0b013e31802b4993 17195809

[mgg31453-bib-0009] Ji, L. D. , Li, J. Y. , Yao, B. B. , Cai, X. B. , Shen, Q. J. , & Xu, J. (2017). Are genetic polymorphisms in the renin‐angiotensin‐aldosterone system associated with essential hypertension? Evidence from genome‐wide association studies. Journal of Human Hypertension, 31(11), 695–698. 10.1038/jhh.2017.29 28425437

[mgg31453-bib-0010] Karaca, E. , Yuregir, O. O. , Bozdogan, S. T. , Aslan, H. , Pehlivan, D. , Jhangiani, S. N. , … Lupski, J. R. (2015). Rare variants in the notch signaling pathway describe a novel type of autosomal recessive Klippel‐Feil syndrome. American Journal of Medical Genetics. Part A, 167A(11), 2795–2799. 10.1002/ajmg.a.37263 26238661 PMC4837953

[mgg31453-bib-0011] Kircher, M. , Witten, D. M. , Jain, P. , O'Roak, B. J. , Cooper, G. M. , & Shendure, J. (2014). A general framework for estimating the relative pathogenicity of human genetic variants. Nature Genetics, 46(3), 310–315. 10.1038/ng.2892 24487276 PMC3992975

[mgg31453-bib-0012] Lin, M. , Zhao, S. , Liu, G. , Huang, Y. , Yu, C. , Zhao, Y. , … Wu, N. (2020). Identification of novel FBN1 variations implicated in congenital scoliosis. Journal of Human Genetics, 65(3), 221–230. 10.1038/s10038-019-0698-x 31827250 PMC6983459

[mgg31453-bib-0013] Liu, J. , Wu, N. , Yang, N. , Takeda, K. , Chen, W. , Li, W. , … Qiu, G. (2019). TBX6‐associated congenital scoliosis (TACS) as a clinically distinguishable subtype of congenital scoliosis: further evidence supporting the compound inheritance and TBX6 gene dosage model. Genetics in Medicine, 21(7), 1548–1558. 10.1038/s41436-018-0377-x 30636772 PMC6659397

[mgg31453-bib-0014] Maguire, R. J. , Isaacs, H. V. , & Pownall, M. E. (2012). Early transcriptional targets of MyoD link myogenesis and somitogenesis. Developmental Biology, 371(2), 256–268. 10.1016/j.ydbio.2012.08.027 22954963

[mgg31453-bib-0015] Morimoto, M. , Takahashi, Y. , Endo, M. , & Saga, Y. (2005). The Mesp2 transcription factor establishes segmental borders by suppressing Notch activity. Nature, 435(7040), 354–359. 10.1038/nature03591 15902259

[mgg31453-bib-0016] Nakajima, Y. , Morimoto, M. , Takahashi, Y. , Koseki, H. , & Saga, Y. (2006). Identification of Epha4 enhancer required for segmental expression and the regulation by Mesp2. Development, 133(13), 2517–2525. 10.1242/dev.02422 16728472

[mgg31453-bib-0017] Oginuma, M. , Niwa, Y. , Chapman, D. L. , & Saga, Y. (2008). Mesp2 and Tbx6 cooperatively create periodic patterns coupled with the clock machinery during mouse somitogenesis. Development, 135(15), 2555–2562. 10.1242/dev.019877 18579680

[mgg31453-bib-0018] Schaffer, A. A. (2013). Digenic inheritance in medical genetics. Journal of Medical Genetics, 50(10), 641–652. 10.1136/jmedgenet-2013-101713 23785127 PMC3778050

[mgg31453-bib-0019] Shen, J. , Wang, Z. , Liu, J. , Xue, X. , & Qiu, G. (2013). Abnormalities associated with congenital scoliosis: a retrospective study of 226 Chinese surgical cases. Spine (Phila Pa 1976), 38(10), 814–818. 10.1097/BRS.0b013e31827ed125 23197014

[mgg31453-bib-0020] Sparrow, D. B. , McInerney‐Leo, A. , Gucev, Z. S. , Gardiner, B. , Marshall, M. , Leo, P. J. , … Dunwoodie, S. L. (2013). Autosomal dominant spondylocostal dysostosis is caused by mutation in TBX6. Human Molecular Genetics, 22(8), 1625–1631. 10.1093/hmg/ddt012 23335591

[mgg31453-bib-0021] Stamataki, D. , Kastrinaki, M. , Mankoo, B. S. , Pachnis, V. , & Karagogeos, D. (2001). Homeodomain proteins Mox1 and Mox2 associate with Pax1 and Pax3 transcription factors. FEBS Letters, 499(3), 274–278. 10.1016/s0014-5793(01)02556-x 11423130

[mgg31453-bib-0022] Takahashi, J. , Ohbayashi, A. , Oginuma, M. , Saito, D. , Mochizuki, A. , Saga, Y. , & Takada, S. (2010). Analysis of Ripply1/2‐deficient mouse embryos reveals a mechanism underlying the rostro‐caudal patterning within a somite. Developmental Biology, 342(2), 134–145. 10.1016/j.ydbio.2010.03.015 20346937

[mgg31453-bib-0023] Takahashi, Y. , Koizumi, K. , Takagi, A. , Kitajima, S. , Inoue, T. , Koseki, H. , & Saga, Y. (2000). Mesp2 initiates somite segmentation through the Notch signalling pathway. Nature Genetics, 25(4), 390–396. 10.1038/78062 10932180

[mgg31453-bib-0024] Wang, K. , Zhao, S. , Liu, B. , Zhang, Q. , Li, Y. , Liu, J. , … Wu, N. (2018). Perturbations of BMP/TGF‐beta and VEGF/VEGFR signalling pathways in non‐syndromic sporadic brain arteriovenous malformations (BAVM). Journal of Medical Genetics, 55(10), 675–684. 10.1136/jmedgenet-2017-105224 30120215 PMC6161649

[mgg31453-bib-0025] Windner, S. E. , Doris, R. A. , Ferguson, C. M. , Nelson, A. C. , Valentin, G. , Tan, H. , … Devoto, S. H. (2015). Tbx6, Mesp‐b and Ripply1 regulate the onset of skeletal myogenesis in zebrafish. Development, 142(6), 1159–1168. 10.1242/dev.113431 25725067 PMC4360180

[mgg31453-bib-0026] Wu, N. , Ming, X. , Xiao, J. , Wu, Z. , Chen, X. , Shinawi, M. , … Zhang, F. (2015). TBX6 null variants and a common hypomorphic allele in congenital scoliosis. New England Journal of Medicine, 372(4), 341–350. 10.1056/NEJMoa1406829 25564734 PMC4326244

[mgg31453-bib-0027] Wu, N. , Wang, L. , Hu, J. , Zhao, S. , Liu, B. , Li, Y. , … Qiu, G. (2019). A recurrent rare SOX9 variant (M469V) is associated with congenital vertebral malformations. Current Gene Therapy, 19(4), 242–247. 10.2174/1566523219666190924120307 31549955

[mgg31453-bib-0028] Yang, N. , Wu, N. , Zhang, L. , Zhao, Y. , Liu, J. , Liang, X. , … Zhang, F. (2019). TBX6 compound inheritance leads to congenital vertebral malformations in humans and mice. Human Molecular Genetics, 28(4), 539–547. 10.1093/hmg/ddy358 30307510 PMC6489408

[mgg31453-bib-0029] Yasuhiko, Y. , Haraguchi, S. , Kitajima, S. , Takahashi, Y. , Kanno, J. , & Saga, Y. (2006). Tbx6‐mediated Notch signaling controls somite‐specific Mesp2 expression. Proceedings of the National Academy of Sciences of the United States of America, 103(10), 3651–3656. 10.1073/pnas.0508238103 16505380 PMC1450137

[mgg31453-bib-0030] Zhao, S. , Zhang, Y. , Chen, W. , Li, W. , Wang, S. , Wang, L. , … Wu, N. (2020). Diagnostic yield and clinical impact of exome sequencing in early‐onset scoliosis (EOS). Journal of Medical Genetics. 10.1136/jmedgenet-2019-106823 PMC7802082 32381727

